# Phenotypic plasticity in visual opsin gene expression: a meta-analysis in teleost fish

**DOI:** 10.1242/jeb.250332

**Published:** 2025-06-30

**Authors:** César Bertinetti, Julián Torres-Dowdall

**Affiliations:** Department of Biological Sciences, University of Notre Dame, Notre Dame, IN 46556, USA

**Keywords:** Visual ecology, Sensory adaptations, Color vision plasticity, Spectral tuning

## Abstract

Phenotypic plasticity in visual opsin gene expression enables teleost fish to adjust their spectral sensitivity in response to environmental variability, yet the magnitude and drivers of this plasticity remain unresolved. We conducted a meta-analysis of 573 effect sizes from 36 studies to assess the prevalence and strength of opsin gene expression plasticity across teleost fishes, considering stimulus type (internal versus external) and timing of exposure (acute versus developmental). Plasticity of opsin gene expression was widespread and generally strong, with internal stimuli (e.g. hormonal changes) eliciting larger and more consistent responses than external stimuli (e.g. light conditions), particularly in the red-sensitive *lws* and UV-sensitive *sws1* opsin genes. Phylogenetic analysis revealed no significant evolutionary constraints on opsin plasticity, suggesting that the capacity for visual system modulation is broadly distributed across teleost lineages. Our findings highlight the need for future studies to integrate behavioral, molecular and ecological data to evaluate the adaptive significance of opsin plasticity and its role in shaping visual performance under changing environmental conditions.

## Teaser

**Highlighted Article:** A meta-analysis reveals widespread phenotypic plasticity in opsin gene expression among teleost fish and its common drivers, highlighting key future research areas to achieve an integrative understanding of opsin plasticity.

## INTRODUCTION

Phenotypic plasticity is a key evolutionary mechanism that enables organisms to modulate their phenotypes in response to environmental conditions without genetic changes (Schlichting and Pigliucci, 1998; Stearns, 1989; Whitman and Agrawal, 2009). This flexibility allows species to alter their behavioral, physiological and morphological traits in response to stimuli, potentially increasing their chances of survival and/or reproduction in heterogeneous habitats and, thus, their fitness (Pigliucci, 1996; Scheiner and DeWitt, 2004). Therefore, understanding the role of phenotypic plasticity is critical for predicting organismal responses to varying environmental conditions, particularly under the current threat of climate change and rapid anthropogenic shifts in natural ecosystems (Fox et al., 2019; Scheiner et al., 2020).

Aquatic ecosystems represent fluctuating habitats that vary dynamically in terms of abiotic properties. For instance, light composition in underwater habitats is highly variable compared with that in terrestrial ecosystems (Lythgoe, 1979; Sabbah et al., 2013). These heterogeneous habitats present challenges for visually oriented organisms because of their fluctuating nature across spatial and temporal scales (Bowmaker, 1990; Johnsen, 2012; Loew and McFarland, 1990; Lythgoe, 1975, 1979). Photic variability is a known driver of visual systems diversity in teleost fish, leading to differences in the number of opsin genes, gene sequences, expression patterns, chromophore usage and photoreceptor properties (Bertinetti et al., 2024b; Cronin et al., 2014; Cummings and Endler, 2018; Munz and McFarland, 1977; Musilova et al., 2021; Torres–Dowdall et al., 2021). While environmental factors, feeding ecology and sexual selection also contribute to visual diversity (Boughman, 2001; Hofmann et al., 2009; Seehausen et al., 2008), this phenotypic variation is not entirely driven by genetic variation, thus underscoring the significance of visual plasticity (Bertinetti et al., 2024a; Fogg et al., 2023; Fuller et al., 2004; Härer et al., 2018; Rennison et al., 2016; Torres-Dowdall et al., 2017). Therefore, phenotypic plasticity seems key for aquatic organisms to dynamically fine-tune visual sensitivity and maintain performance under changing light conditions.

Historically, visual opsin gene expression was viewed through a relatively static lens: one opsin gene corresponded to one visual pigment, which corresponded to one photoreceptor type (Bowmaker, 1990; Lythgoe, 1979; Walls, 1942). This fixed perspective shifted dramatically in the early 21st century with a surge of studies revealing that opsin gene expression is plastic (Cheng and Flamarique, 2004; Fuller et al., 2005). The evidence for opsin gene expression plasticity meant that morphologically identical photoreceptor cells (e.g. single cones) can switch the opsin gene they express, thereby regulating their spectral phenotype (e.g. shifting from ultraviolet to blue sensitivity; Cheng et al., 2006; Cheng and Flamarique, 2004; Dalton et al., 2014). Differential expression of opsin genes in this context has been interpreted as likely reflecting changes in the relative abundance of spectral cone types or a shift in the opsins expressed within individual photoreceptors (Cheng and Flamarique, 2007; Dalton et al., 2014; Flamarique et al., 2013; Torres-Dowdall et al., 2017). Opsin gene expression changes might also reflect the density of visual pigments found in a given photoreceptor, or morphological differences; that is, larger photoreceptors containing more opsin protein (Fuller and Claricoates, 2011; Kröger et al., 1999). Theoretically, all this visual plasticity mirrors the high spectral heterogeneity of aquatic environments, providing a reliable mechanism for fish to fine-tune their visual sensitivity to ongoing ecological demands (Fuller and Claricoates, 2011; Härer et al., 2019; Shaughnessy and Cortesi, 2024). Moreover, the finding that opsin gene expression varies across the day in some species contributed to a view of opsin gene expression as highly dynamic (Johnson et al., 2013; Yourick et al., 2019). However, those examples contrast with numerous studies documenting minimal or absent plasticity in response to light stimuli (e.g. Flamarique et al., 2013; Stieb et al., 2016; Valen et al., 2018). Hence, the frequency and magnitude of opsin expression plasticity and how this relates to certain phylogenetic groups remains unclear.

The diversity of teleost visual systems and their plasticity has stimulated a lot of research, much of it focusing on opsin gene expression. Plasticity in opsin gene expression is associated with seasonal or episodic environmental fluctuations (Foster et al., 2025; Valen et al., 2018), developmental transitions (Cheng et al., 2006; Lupše et al., 2022) and reproductive cycles (Li et al., 2022; Shao et al., 2014). For instance, seasonal migration of diadromous fish often correlates with changes in opsin gene expression to adjust to novel photic conditions (Bowmaker, 1995; Thomson-Laing et al., 2018). Similarly, opsin gene expression patterns change during development, usually linked to hormonally driven maturation from early-life planktivory to diverse adult foraging styles (Cheng and Flamarique, 2004; Temple et al., 2008). Reproductive state influenced by sex hormones might also correlate with plastic changes in opsin gene expression to optimize mate recognition using visual cues (Brock et al., 2018; Butler and Maruska, 2021). Although the consequences of opsin plasticity for visual sensitivity remain often untested, its prevalence in non-manipulated natural systems suggests a relevant role of opsin gene expression plasticity within the sensory biology of teleost fish. Given the multiple factors influencing opsin gene expression, studies have predominantly taken a mechanistic approach, manipulating single stimuli under laboratory conditions to measure whether and which opsin genes exhibit plasticity.

The literature on opsin expression plasticity is complex and diverse, reflecting a variety of experimental approaches. Some studies have investigated the overall response of the visual system to environmental manipulations (e.g. Kröger, 2013; Kröger and Fernald, 1994; Tiarks et al., 2024; Wagner and Kröger, 2005), while others have focused specifically on the plasticity of opsin gene expression (e.g. Fogg et al., 2023; Fuller et al., 2005; Härer et al., 2017; Hofmann et al., 2010; Nandamuri et al., 2017). Methodological differences are prominent, with some studies emphasizing the effects of internal cues, such as hormonal changes (e.g. Karagic et al., 2022; Temple et al., 2008; Volkov et al., 2020), and other studies examining responses to external stimuli, such as light conditions or photic environments (e.g. Härer et al., 2017; Kranz et al., 2018; Schweikert and Grace, 2018; Wilwert et al., 2023). Few studies have used mixed designs incorporating both internal and external stimuli (e.g. Schreiner et al., 2022; Torres-Dowdall et al., 2024). What remains unclear is whether internal and external stimuli exert similar or distinct influences on visual system plasticity.

Furthermore, research on opsin gene expression plasticity varies not only in the type of stimuli but also in the timing and duration of these stimuli. Two common experimental designs have been used to simulate responses triggered by developmental plasticity versus rapid reversible plasticity (i.e. labile plasticity sensu Lande, 2019; or phenotypic flexibility sensu Piersma and Drent, 2003). To study developmental plasticity, researchers have used prolonged rearing conditions in the presence of distinct stimuli, for example, hormone exposure versus control, or white light versus red light (Frau et al., 2022; Härer et al., 2017; Karagic et al., 2022). Alternatively, acute responses due to rapid, short-term environmental changes (e.g. sudden exposure to novel light conditions) have been used to study reversible plasticity (e.g. Chang and Yan, 2019; Härer et al., 2019; Shao et al., 2014). However, it remains unclear to what extent the timing and duration of stimuli influence opsin gene expression plasticity.

The methodological diversity described above complicates the comprehensive understanding of the ubiquity of opsin gene expression plasticity across teleost fish and the main stimuli influencing such plastic responses. Here, we compile data on opsin gene expression plasticity, the most extensively quantified aspect of visual system plasticity in fish. We then perform a meta-analysis to address several questions: (1) Is there substantial evidence of plasticity in opsin gene expression among teleost fish? (2) Does plasticity in opsin gene expression vary depending on the type of stimulus (internal versus external)? (3) Does the timing of exposure, whether acute or developmental, influence plastic responses? Through this analysis, we highlight emerging patterns and identify relevant areas for future research. We emphasize the need for further investigation into the adaptive value of opsin expression plasticity, the consequences of opsin plasticity for visual sensitivity, the temporal dynamics of plasticity, and the integration of plastic responses across different visual system components. Addressing these areas will enhance our understanding of the evolutionary and functional implications of visual plasticity in teleost fish.

## MATERIALS AND METHODS

### Literature search and data collection

To summarize the evidence of phenotypic plasticity in opsin gene expression in fish, we performed a literature search of peer-reviewed publications. The ISI Web of Science and the NCBI PubMed databases were searched in November 2023 and January 2025 using the keywords plastic+opsin+fish, hormone+opsin+fish and fish+visual plasticity, which delivered 121 studies. A total of 36 studies that reported quantitative estimates of opsin gene expression in fish retinas were retained (Table S1). Overall, RT-qPCR was the most used opsin gene quantification technique, used by 75% of the studies, followed by RNA-seq (11%), RNA *in-situ* hybridization (8%) and digital PCR (6%). However, quantification methods did not account for differences in effect sizes among studies (*Q*_M_=0.4256; d.f.=3; *P*=0.935). The references from the 36 studies included were also searched for additional publications. All studies that compared opsin gene expression between distinct light conditions or hormone exposure, either using manipulative treatments or between laboratory-reared and wild-caught individuals were included (Table S1). Only one study was excluded because treatments simultaneously combined both light and temperature exposure (Shimmura et al., 2017). We tested and failed to find differences in effect sizes between laboratory or field-based experiments (*Q*_M_=1.465; d.f.=1; *P*=0.221, Figs S1, S2), with 88% studies being performed under laboratory conditions, hence, these factors were not included in our downstream analyses.

Visual opsin genes were grouped according to their respective ancestral gene class: namely, ultraviolet-sensitive *sws1*, blue-sensitive *sws2,* dim-light sensitive *rh1*, green-sensitive *rh2* and red-sensitive *lws* (Yokoyama, 2000). These five visual opsin gene classes predating the Neoteleostei ancestor have resulted in the wide diversity of visual opsin genes observed in extant teleost fishes (Lin et al., 2017; Rennison et al., 2012). Owing to multiple tandem and whole genome duplications paired with gene conversions and gene losses, the number of visual opsin genes present in a teleost genome varies widely from one to 40, with marked differences in copy number even within the same gene class (Cortesi et al., 2015; Musilova et al., 2019). Since opsin gene paralogs might vary in their expression patterns, we also tested for differences in plasticity within gene classes by ranking the most- and least-plastic opsin gene within each class within each experimental unit. Given the complex phylogenetic relationship and inconsistent nomenclature of opsin gene orthologs, our test was designed to identify between-paralog plasticity, independently of paralog identity. If variation in plastic responses among paralogs is high, we would expect the most plastic paralog to differ significantly from the least plastic one in its plastic capacity. Alternatively, if all paralogs show intermediate levels of plasticity, little variation in plastic responses within each opsin gene class could be expected.

Additionally, the experimental design used by studies was categorized based on the stimulus and type of exposure. Stimuli were recorded as either ‘internal’, such as hormone-induced changes (e.g. thyroxine exposure), or ‘external’, such as effects of light treatment (e.g. red versus white light). Experimental design was categorized as ‘developmental’, if treatment was experienced through an individual's lifespan, or as ‘acute’, if the individual was exposed to a condition different from the one it was raised in. When not explicitly reported in the source study, values of opsin gene expression were extracted from figures using WebPlotDigitizer v4 (Rohatgi, 2017).

### Meta-analysis and phylogenetic signal

To compare the effect sizes of phenotypic responses in teleost fish across studies and to estimate the heterogeneity among studies, we performed a meta-analysis using the *metafor* package (https://CRAN.R-project.org/package=metafor; Viechtbauer, 2010) in R (r-project.org). First, the standardized mean differences (SMD) in opsin gene expression between treatments for all studies were calculated using the *escalc* function. SMD were calculated as the mean difference between two experimental groups divided by their pooled standard deviation while correcting for small sample size bias (Hedges, 1981; Viechtbauer, 2010). The absolute SMD (|SMD|) was used as an estimate of the overall change in opsin gene expression regardless of the directionality of the change (Palacio-López et al., 2015). We used absolute values for two reasons: (1) to quantify the overall plastic response, regardless of the direction of the phenotypic change, and (2) to avoid the confounding effect of some opsin genes being upregulated and others being downregulated in response to the same treatment within an individual. The mean estimates and the variability among observed effect sizes were computed using multilevel linear mixed-effects models based on restricted maximum likelihood implemented with the *rma.mv* function (Viechtbauer, 2010).

To control for the non-independence of estimates, we included random effects that accounted for data points from the same study, different experiments within the same study, species identity, and the phylogenetic relationship between species (Table S2). Phylogenetic trees were generated from Open Tree of Life using the *rotl* package (https://CRAN.R-project.org/package=rotl; Hinchliff et al., 2015; Michonneau et al., 2016) and used to compute the phylogenetic variance-covariance matrix in the *ape* package (https://CRAN.R-project.org/package=ape; Paradis and Schliep, 2019). The effect of moderators, stimulus type (e.g. interval versus external) and timing/duration (developmental versus acute) was performed using an omnibus test followed by a Wald-type chi-square test (Viechtbauer, 2010). To quantify the effect of shared phylogeny on opsin gene expression plasticity, we quantified the phylogenetic contribution, Pagel's λ, of absolute effect sizes using the *phylosignal* package (https://CRAN.R-project.org/package=phylosignal; Keck et al., 2016; Pagel, 1999). The strength of effect sizes is reported as weak (|SMD|<0.5), moderate (|SMD|=0.5–0.8) or strong (|SMD|>0.8) following the consensus on their interpretation (Andrade, 2020; Cohen, 1988). Estimates of variability among studies are reported using *I^2^*, i.e. heterogeneity, as a measure of the relative variance between studies relative to the overall variance (Nakagawa and Santos, 2012; Senior et al., 2016). Similarly, the degree of heterogeneity is reported as small (25%), moderate (50%) or high (>75%) following Higgins and Thompson (2002). The influence of publication bias on effect size estimates was analysed using both funnel plots and multi-level regression as recommended by Nakagawa et al. (2022). We did not find evidence of publication bias within the literature surveyed, no significant small study effects (*t*=−1.11, d.f.=567, *P*=0.266) and no time-lag bias/decline effects over time (*t*=1.51, d.f.=567, *P*=0.131; Figs S3, S4).

## RESULTS

### Phenotypic plasticity in opsin gene expression is widespread and strong

**Fig. 1.**
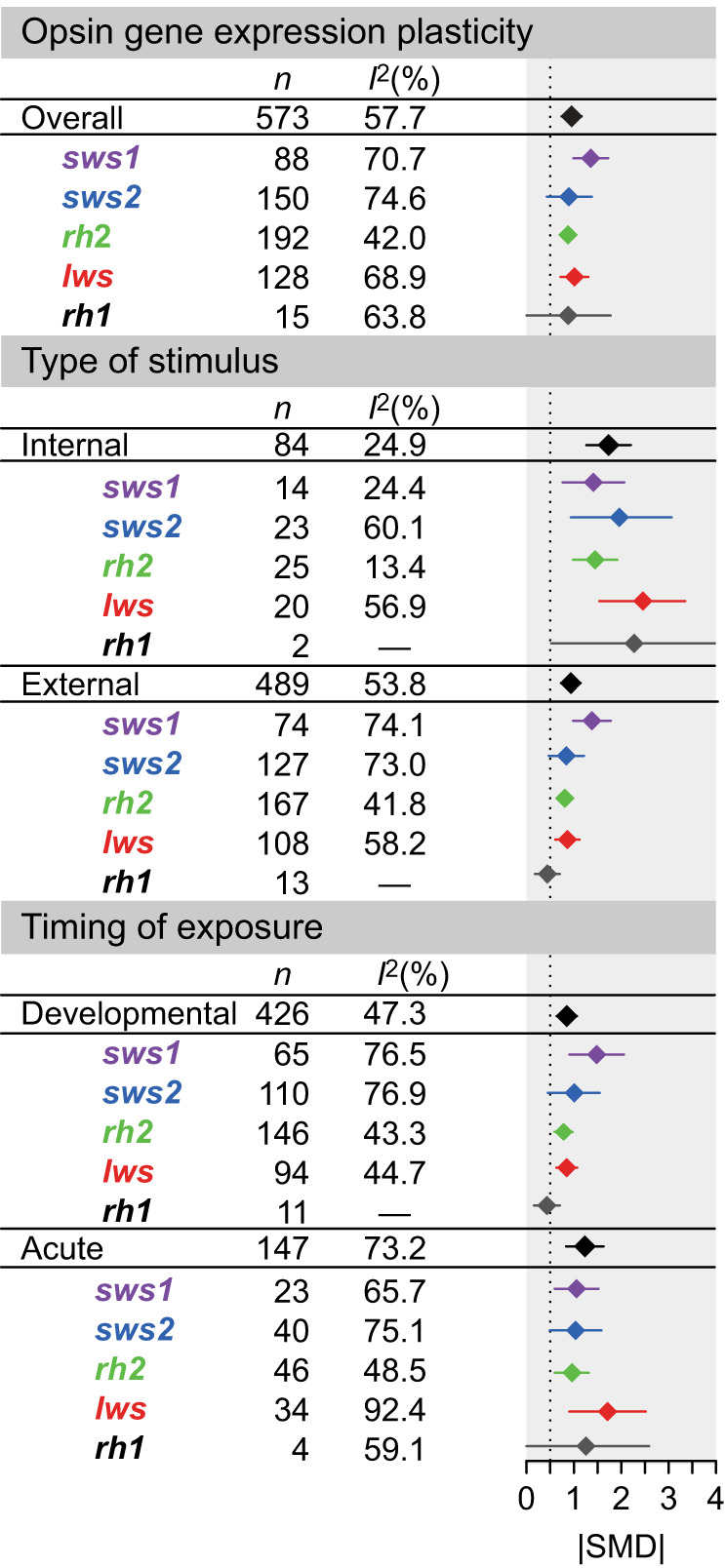
**Absolute standardized mean differences (|SMD|) of different experimental conditions on the five major classes of visual opsin genes.** Estimates were obtained using phylogenetically controlled multivariate meta-analysis models based on restricted maximum likelihood (Viechtbauer, 2010). Diamonds and bars depict mean estimates and 95% confidence intervals.

**Fig. 2.**
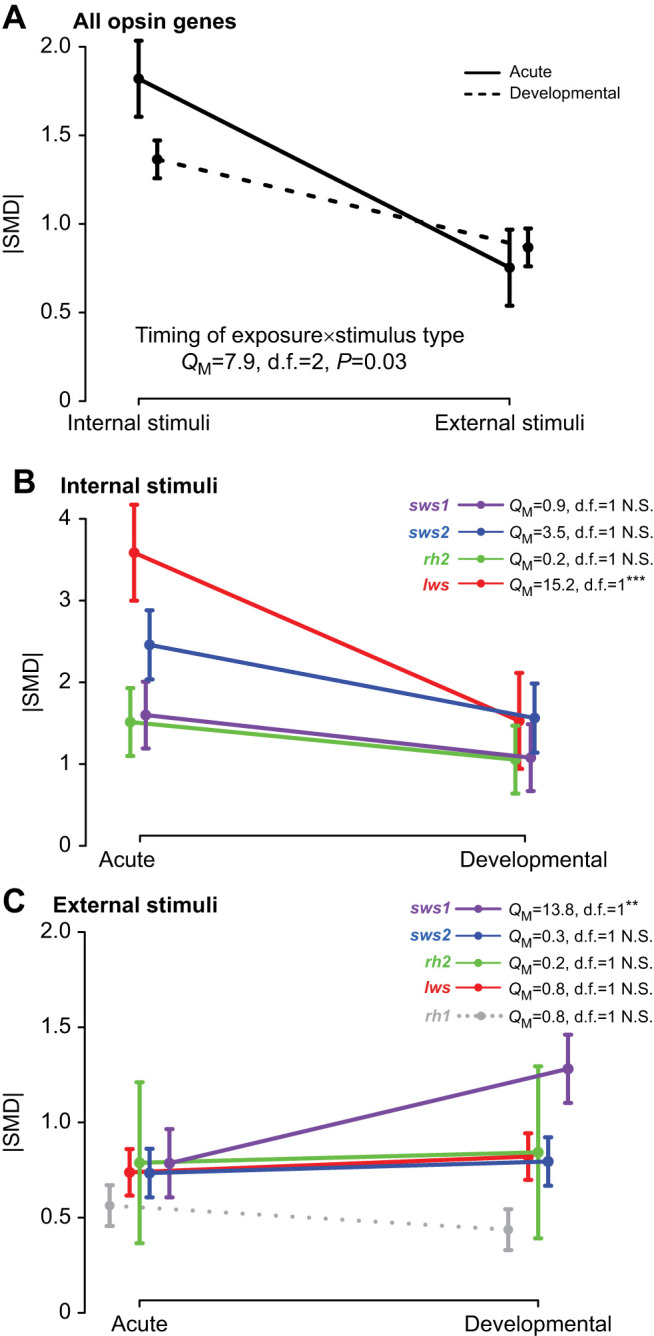
**Absolute standardized mean differences (**|**SMD**|**) of opsin gene expression plasticity in response to different stimuli type and timing of exposure.** (A) Interaction between timing of exposure and stimulus type. (B) Internal and (C) external stimuli elicit distinct plastic responses among opsin genes. Dot and error bars represent mean and 95% confidence intervals. *P*-values estimated using Wald-type chi-square test and adjusted for multiple comparisons using false-discovery rate. ***P<0.001; **P<0.01; N.S., not significant.

Overall, 573 effect sizes of plasticity in opsin gene expression were obtained from 36 studies. The meta-analysis revealed widespread strong phenotypic plasticity in opsin gene expression but heterogeneity among studies was moderate (*Q*_E_=1570.6; d.f.=569; *P*<0.001; Fig. 1, Table S2). Effect sizes were significantly influenced by an interaction between the timing of exposure and stimulus type (Fig. 2, Table S3). The interaction was evidenced by plastic responses due to internal stimuli for acute exposure being higher than ones with developmental exposure (*Q*_M_=3.8727; d.f.=1; *P*=0.048, Fig. 2A). However, plastic responses due to external stimuli did not differ significantly between acute and developmental exposure (*Q*_M_=2.6475, d.f.=1, *P*=0.104).

Across all opsins, 66.5% of effect sizes were moderate to high (|SMD|>0.5), indicating that plasticity is generally robust, though this proportion varied by gene class, with the lowest proportion for *rh1* (40%) and the highest for *sws1* (78%), followed by *sws2* (66%), *rh2* (64%) and *lws* (64%; Fig. S5). While most opsin genes did not differ significantly in their gene expression plasticity in response to acute or developmental exposure, there were two exceptions: the red-sensitive opsin *lws* showed increased plasticity in response to acute hormonal exposure (Fig. 2B), whereas UV-sensitive *sws1* exhibited greater plasticity under developmental exposure to external stimuli (Fig. 2C). One potential explanation for this finding is the number of paralogs, as *sws2* and *rh2* tend to have more paralogs within species. In these cases, the overall plastic response may be dampened if some paralogs are highly plastic while others are not, masking stronger individual responses when averaged. To test this, we analyzed plasticity within the three opsin gene classes containing multiple paralogs (*sws2*, *rh2*, and *lws*). All paralogs showed moderate to high plasticity (|SMD|>0.5) and significant differences were found between the most and least plastic paralogs within each class (Fig. S6). However, when comparing the most or least plastic paralogs across gene classes (e.g. most plastic *rh2* versus most plastic *lws*), no significant differences in plasticity were found (Fig. S6). Together, these results indicate that the range of plastic responses is similar across opsins, regardless of paralog number. Thus, higher plasticity observed for *lws* under acute hormonal exposure and for *sws1* under developmental exposure to external stimuli is unlikely to be explained by variation in paralog number alone.

Additionally, plasticity in opsin gene expression in response to internal stimuli exhibited larger effect sizes and smaller heterogeneity than in response to external stimuli (Fig. 1). Most effect sizes (87%) were moderate to high across opsins when exposed to internal stimuli (*sws1*=86%, *sws2*=87%, *rh2*=84%, *lws*=90% and *rh1*=100%). In contrast, external stimuli tend to induce less pronounced plastic responses with only 63% of effect sizes being above 0.5 (|SMD|>0.5), except for *sws1*=77% (*sws2*=62%, *rh2*=62%, *lws*=60% and *rh1*=31%)*.* Timing of exposure did not affect the magnitude and heterogeneity of effect sizes in opsin gene expression plasticity (Fig. 1). The percentage of effect sizes above 0.5 was 66% for developmental treatments (*sws1*=80%, *sws2*=65%, *rh2*=62%, *lws*=66% and *rh1*=27%) and 68% for acute treatments (*sws1*=74%, *sws2*=67%, *rh2*=72%, *lws*=62% and *rh1*=75%).

### Opsin gene expression plasticity varies across taxa but is not phylogenetically constrained

**Fig. 3.**
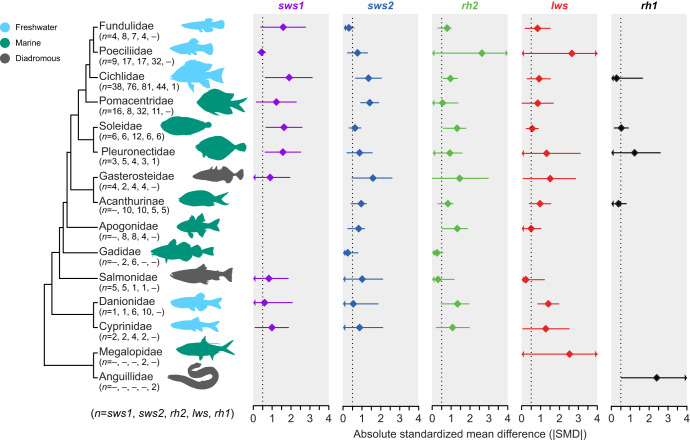
**Meta-analysis of the absolute effect sizes of different experimental conditions across teleost families.** Estimates were obtained using phylogenetically controlled multivariate meta-analysis models based on restricted maximum likelihood (Viechtbauer, 2010). Diamonds and bars depict mean estimates and 95% confidence intervals, respectively. Confidence intervals extending beyond the figure range are represented with an arrow. When *n*=1, 95% confidence intervals represent the within-study uncertainty (1.96 times the squared sampling variance of the effect size).

A total of 37 species of teleost fish from 15 families were included in this study (Fig. 3). Based on habitat type, freshwater taxa were represented most frequently (62%), followed by marine (29%) and diadromous species (8%). Cichlidae had the largest effect sizes reported in the literature (40%) followed by Poecilidae (14%) and Pomacentridae (12%). The predominance of certain taxonomic groups in the study of opsin gene expression plasticity is further evidenced when looking at habitat type, where Cichlidae and Poecilidae contribute to 64% and 22% of effect sizes from freshwater species, respectively. A similar trend was observed in marine habitats, where four families account for over 96% of the effect sizes: Pomacentridae (46%), Soleidae (22%), Acanthuridae (16%) and Apogonidae (12%). All families had at least one opsin that showed moderate to strong plastic responses (|SMD|>0.5, Fig. 3). However, three species out of 37 showed only small effect sizes (*Dascyllus aruanus*, *Oncorhynchus tshawytscha*, *Pundamilia pundamilia*). Furthermore, the phylogenetic signal did not account for differences in the mean effect sizes among taxa, with Pagel's λ∼0, suggesting the widespread, phylogeny-independent presence of opsin gene expression plasticity among teleost fish (Figs S7, S8). The plasticity of individual visual opsin genes did not exhibit any phylogenetic signal either (Fig. S9).

## DISCUSSION

Phenotypic plasticity, the ability of organisms to adjust their traits in response to environmental changes, plays a critical role in survival and reproduction (Bradshaw, 1965; Ghalambor et al., 2007; West-Eberhard, 2003). Sensory systems, including vision, must constantly recalibrate their sensitivity to extract relevant signals while minimizing noise (Cronin et al., 2014; Vorobyev and Osorio, 1998). Teleost fishes, which occupy aquatic environments with pronounced variability in photon availability and spectral composition, provide a powerful model for studying visual plasticity (Kirk, 1985; Lythgoe, 1979; Shaughnessy and Cortesi, 2024). Their diverse habitats allow for testing links among opsin gene expression, phenotype and function across a broad range of ecological conditions. Our meta-analysis of opsin gene expression reveals that teleost visual systems generally exhibit moderate to high plastic responses, independently of phylogenetic relatedness. However, individual opsin genes vary in their plasticity and respond differentially depending on the type of stimulus and the timing of exposure. Based on these findings and a review of the literature, we identify key knowledge gaps and propose future research directions for advancing our understanding of sensory plasticity (Box 1).

Box 1. Open questions and future directions **Does opsin plasticity affect visual sensitivity?**

Most studies on opsin plasticity are descriptive or correlate gene expression with external factors, assuming that changes in opsin gene expression reflect spectral cone type abundance. However, the implications of opsin gene expression for visual sensitivity lack experimental confirmation in most systems. Addressing this requires physiological and behavioral studies combined with gene expression and protein quantification (e.g. Escobar-Camacho et al., 2017; Sabbah et al., 2010; Smith et al., 2012).

**What is the adaptive significance of opsin plasticity?**

Testing the adaptive value of opsin plasticity in ecologically relevant settings is critical. This requires integrating transcriptomic data with behavioral assays to assess the contributions of opsin plasticity to performance or fitness (e.g. Novales Flamarique, 2016).

**Is opsin plasticity age-dependent?**

Similar molecular pathways may underlie both developmental and plastic responses. While opsin plasticity appears common, opsin genes vary in their responsiveness during development. Future work should use experimental designs that combine stimulus types and timing of exposure (e.g. Schreiner et al., 2022; Torres-Dowdall et al., 2024). Standardized light treatments across species and dose response hormonal curves are needed to ensure that realistic stimuli are tested (e.g. Thomson-Laing et al., 2018).

**What are the temporal dynamics of opsin plasticity?**

Understanding how rates of phenotypic change match the frequency and speed of ecologically realistic stimuli is essential (Burton et al., 2022). This requires multiple iterative measurements of opsin gene expression following environmental changes, ideally using long-term environmental data and phenotypic traits from wild populations (e.g. Foster et al., 2025).

**Is opsin plasticity modular or integrated?**

Plastic responses of visual system components may be modular (independently regulated) or integrated (co-regulated across multiple traits). Addressing this requires measuring multiple traits such as lens transmittance, retinal morphology, photoreceptor composition, retinal ganglion cell density, and chromophore usage (e.g. Bertinetti et al., 2025; Torres-Dowdall et al., 2017).

**How does opsin plasticity influence ecological communities?**

Opsin plasticity in response to environmental changes could influence ecosystem dynamics. Addressing this requires linking patterns of plasticity across multiple co-existing species to the heterogeneity of their environments (Härer et al., 2018; e.g. Stieb et al., 2016).

Our meta-analysis reveals that opsin gene expression plasticity is widespread among teleost fishes. Across all visual opsin genes, moderate (66%) and strong (51%) effect sizes prevailed, and these patterns were not explained by phylogenetic relatedness (Figs S5, S9). Notably, plasticity varied among opsin gene classes: *sws1* and *lws* showing higher plastic responses than *sws2*, *rh2 and rh1* (Figs S1, S2). The UV-sensitive opsin *sws1* showed the highest plastic response to light stimuli experienced during development (Fig. 2C). UV sensitivity plays a key role in the ontogenetic dietary shifts of fish, thus favoring the developmental plasticity of *sws1* (Loew et al., 1993; Flamarique, 2013). Supporting our findings, Lupše et al. (2022) also reported that *sws1* was the only cone opsin to show consistent developmental expression changes across multiple fish species. In contrast, the red-sensitive opsin *lws* showed the highest plastic response to internal stimuli with acute exposure (Fig. 2B). *lws* mediates mating preferences in several fish species and thus might be particularly sensitive to hormonal fluctuations involved in visual mating signals and reproductive behavior (Brock et al., 2018; Shao et al., 2014; Smith et al., 2012). These functional associations suggest that opsin-specific plasticity patterns may reflect ecological relevance and task-specific demands. However, the relationship between gene expression plasticity, visual sensitivity and behavioral performance remains poorly understood. Studies assessing individual opsin functions within ecologically realistic contexts that link gene expression with organismal performance are needed to advance the field of visual ecology (Box 1).

The increased plasticity in *sws1* and *lws* compared with other opsin classes may reflect tuning mechanisms to the spectral regions that experience the greatest variability in aquatic environments. These genes encode visual pigments sensitive to the boundaries of the visible spectrum – UV for *sws1* and red/far-red for *lws* (Yokoyama, 2000). Owing to the spectral absorbance properties of water and the influence of dissolved substances such as inorganic salts, organic matter and phytoplankton, these boundary regions (<400 nm and >630 nm) are the most variable (Kirk, 1983; Mobley, 1994). Consequently, natural selection may favor greater phenotypic variation at these spectral extremes. For instance, persistent selection at these spectral regions might promote elevated genetic divergence in *lws* and *sws1* (Hofmann et al., 2009). Conversely, an alternative – but not mutually exclusive – hypothesis suggests that spectral tuning at the boundaries of the visible range is primarily achieved through plastic regulation of opsin gene expression (e.g. this meta-analysis; Bloch, 2015). Our findings support this later view, as *sws1* and *lws* exhibited highest levels of plasticity (Fig. 2). Moreover, high plasticity may be undesirable for opsins sensitive to intermediate wavelengths (∼400–550 nm), such as s*ws2* and *rh2*, which are spectrally constrained by neighboring opsins and may require more tightly coordinated expression responses. Notably, *sws2* and *rh2* also represent the opsin classes with the highest gene duplication rates and greatest coding sequence divergence among teleosts (Gojobori and Innan, 2009; Irisarri et al., 2018; Musilova and Cortesi, 2023).

Plasticity in opsin gene expression also differed between types of stimuli. Internal stimuli, such as hormonal treatments, elicited stronger and more consistent plastic responses than external stimuli, e.g. environmental light changes (Fig. 1). Stronger plastic responses to internal stimuli may result from artificially high hormone exposure in experimental settings, potentially leading to an ‘overshoot’ effect. While plastic responses to internal stimuli in nature are usually linked to prolonged exposures to ontogenetic, seasonal or reproductive stimuli, laboratory-based manipulative experiments usually elicit acute responses to investigate opsin plasticity mechanisms. Ideally, acute exposure to hormones in laboratory conditions would require a better understanding of hormonal changes in the wild to ensure ecologically relevant concentrations are used (Box 1). The differences in plastic responses to internal and external stimuli support the idea that distinct mechanisms might underlie the modulation of opsin gene expression in each case (Torres-Dowdall et al., 2024). This decoupling of regulatory mechanisms for each stimulus type might facilitate independent optimization of each response (Sambamoorthy and Raman, 2018). Given the high degree of spectral variability in aquatic photic conditions (Cronin et al., 2014; Lythgoe, 1979), external stimuli might provide less reliable cues than internal ones, thus eliciting weaker and more heterogeneous responses (Scheiner, 2018). Remarkably, the timing of exposure to stimuli – either developmental, prolonged or acute, sudden exposure – did not lead to differences in gene expression plasticity in most opsins (Fig. 1 and Fig. 2B). This suggests internal and external stimuli might activate consistent pathway responses across different ages, although the specific responses may differ between the two types of stimuli (Box 1). Further research into the reversibility of opsin gene expression changes during development might be needed to distinguish developmental from acute plastic responses.

Importantly, not only the reversibility of opsin gene expression plasticity but also its temporal dynamics require further attention. While research has predominantly focused on the magnitude of plastic responses, the rate of plastic changes in opsin gene expression has been overlooked. Plastic capacity determines the match between phenotype and environment, but the rate of plasticity influences the duration of the adaptive lag, which is the mismatch between the original phenotype and the new conditions (Burton et al., 2022; Dupont et al., 2024). Although rapid changes in opsin gene expression occurring within hours to days are documented (Fogg et al., 2023; Fuller and Claricoates, 2011; Härer et al., 2017, 2019), few studies have measured the speed of these responses by iteratively quantifying expression post-stimulus (Box 1). Measuring opsin gene expression only once post-stimulus complicates distinguishing transitional and plateau phases of the plastic response, resulting in a failure to estimate both the rate and capacity of plasticity (Burton et al., 2022; Dupont et al., 2024). Since photic fluctuations vary, for example, gradual seasonal shifts versus abrupt storm runoff, understanding rates of plasticity seems key to make inferences about the evolution of opsin gene expression plasticity. Adaptive lags may also be influenced by the time required for gene products to become functional visual pigments (Box 1). Diurnal patterns in opsin expression are particularly relevant in this context, but their mechanisms and implications remain poorly understood (Halstenberg et al., 2005; Johnson et al., 2013; Yourick et al., 2019). Long-term monitoring of photic conditions and continuous phenotypic measurements are essential to address these gaps in understanding the temporal dynamics of opsin plasticity (Box 1). Because measuring opsin gene expression usually requires destructive sampling, individual replicates experiencing the same conditions need to be sampled iteratively to estimate plastic responses over time.

Although opsins are central to color vision, visual plasticity involves multiple system components. Opsin gene expression is only one factor, and changes in other elements may influence or constrain plasticity (Carleton et al., 2020; Torres-Dowdall et al., 2024). For example, teleost fish possess two types of chromophores – vitamin A1- or A2-based – that bind to opsin proteins to form functional visual pigments (Wald, 1939). The type of chromophore can shift the absorbance spectrum of the pigment, thereby affecting visual sensitivity (Enright et al., 2015). Plasticity in chromophore usage has been observed in response to both internal and external stimuli, changing the expression of key enzymes involved in chromophore exchange or the ratio of chromophore types (Bertinetti et al., 2024b; Karagic et al., 2022; Volkov et al., 2020). However, how other components such as photoreceptor morphology, lens transmittance or retinal connectivity respond to novel stimuli and integrate with opsin plasticity remains unclear (Kröger, 2013; Schartau et al., 2009). Integrated responses across the visual system may ensure functionality; for example, fish lacking UV-sensitive *sws1* often have UV-filtering lenses, as decoupling these traits would be maladaptive (Hofmann et al., 2010; Torres-Dowdall et al., 2017). Hence, changes in opsin gene expression might be limited by changes in other visual system components, thus constraining visual plasticity. Conversely, modularity in the visual system might allow independent fine-tuning of components with minimal changes. Investigating whether plastic responses are integrated or modular is key to understanding how they facilitate or constrain plasticity (Box 1). Future research must expand beyond opsin gene expression to provide a comprehensive view of visual plasticity.

Similarly, the ultimate causes of opsin gene expression plasticity remain poorly understood. While proximate causes, mechanisms and developmental aspects have been studied, the functional and adaptive significance of opsin plasticity is often inferred rather than directly tested (Härer et al., 2017; Veen et al., 2017; Wagner and Kröger, 2005). Without linking opsin gene expression plasticity to performance or fitness, the ecological and evolutionary implications remain uncertain (Box 1). Opsin plasticity should theoretically optimize vision for ecologically relevant tasks under fluctuating photic conditions (Carleton et al., 2020; Fuller and Claricoates, 2011; Härer et al., 2017; Shaughnessy and Cortesi, 2024). Comparative studies have provided behavioral evidence of the adaptive benefits of differential opsin gene expression, e.g. UV-sensitive *sws1* enhancing zooplankton foraging (Flamarique, 2016; Rick et al., 2012; Yoshimatsu et al., 2020) or red-sensitive *lws* facilitating mate choice in Malawi cichlids (Smith et al., 2012). Our findings that *sws1* and *lws* are the most plastic opsin gene classes suggest that such plasticity might provide adaptive benefits given their relevance for behavioral tasks. However, specific visual tasks require distinct opsin genes, for example, locating a prey against a background versus choosing a mate based on body coloration. Plasticity in one opsin gene can influence others, creating trade-offs in spectral sensitivity and visual performance. Thus, the adaptive value of opsin plasticity should be examined holistically as a trait rather than focusing on single genes. Integrating transcriptomic, functional and behavioral data is essential for a comprehensive understanding of visual plasticity (Box 1).

Overall, research on visual plasticity has prioritized a phenomenological understanding of opsin gene expression while overlooking its broader implications within an ecological and evolutionary context. Although researchers seem to agree that plastic modulation of opsin gene expression should have ecological consequences, this assumption often lacks empirical validation. In theory, phenotypic plasticity should enable organisms to increase their fitness under heterogeneous conditions (Pigliucci, 1996; Scheiner and DeWitt, 2004). Aquatic habitats face increasing pressure due to major challenges such as invasive species, anthropogenic water pollution or ocean acidification (USGCRP, 2023). Such threats will impact ecosystems by modifying interactions among species with distinct sensory biology, altering visual environments as a result of eutrophication and habitat loss, and reducing the predictability of environmental fluctuations by increasing stochasticity (Bonamour et al., 2019; Caves and Johnsen, 2021; Zanghi and Ioannou, 2025). In this context, visual traits are good candidates to study the role of short-term responses to environmental changes and how these might influence evolutionary trajectories under global change (Box 1). Moreover, aquatic ecosystems represent major economic and societal assets encompassing key biodiversity hotspots (Dudgeon et al., 2006; Roberts et al., 2002). However, the taxonomic bias observed in visual plasticity research limits our predictions about species risks and might obscure the apparent widespread plasticity in opsin gene expression observed in teleost fish (Bertinetti et al., 2025). Therefore, future studies on visual plasticity should seek to expand their focus beyond historically predominant taxonomic groups and incorporate ecology-driven hypotheses into their experimental designs.

To summarize, we show that phenotypic plasticity in opsin gene expression is common among teleost fishes. While significant progress has been made in documenting the plasticity of visual traits, the ecological and evolutionary implications of these changes require further exploration. Research aimed at understanding how changes in opsin gene expression relate to perceptual differences and behavioral performance is still missing in many systems. Our findings highlight the importance of integrating molecular, ecological and behavioral approaches to understand the functional significance of visual plasticity. Bridging the gap between plastic phenotypic responses and their broader ecological and evolutionary consequences is relevant to understanding the link between sensory biology and ecosystem dynamics. Future research should explore the adaptive benefits of visual plasticity and potential constraints, particularly under rapid environmental change. We conclude that there is a need for more comprehensive and ecologically realistic studies that assess the fitness consequences of visual plasticity and explore its evolution across diverse environments.

## Supplementary Material

10.1242/jexbio.250332_sup1 Supplementary information
