# Supplementary material for: Phenotypic plasticity in visual opsin gene expression: a meta-analysis in teleost fish
Source: J Exp Biol. 2025 Jun 30;228(13):jeb250332. doi: 10.1242/jeb.250332 (PMC12268174; doi:10.1242/jeb.250332)
Supplement: Supplementary information [file jexbio-228-250332-s1.pdf]

Table S1. List of publications and their corresponding experimental conditions included in this meta-analysis.

Author	Species	Family	Stimulus	Exposure	Stimulus Type	Method	Site
Bakker et al. (2024)	*Gasterosteus aculeatus*	Gasterosteidae	external	developmental	Photic[a]	RT-qPCR	laboratory[b]
Bolstad and Novales Flamarique (2023)	*Hippoglossus hippoglossus*	Pleuronectidae	external	acute	Spectrum	RT-qPCR	laboratory
Chang and Yan (2019)	*Cyprinella lutrensis*	Cyprinidae	external	acute	Photic	RT-qPCR	laboratory
Chang (2023)	*Gambusia affinis*	Poecilidae	external	acute	Photic	RT-qPCR	laboratory
Gan and Novales Flamarique (2010)	*Oncorhynchus kisutch*	Salmonidae	internal	developmental	T4 (300 µg/L)	RNA ISH	laboratory
Dalton et al. (2015)	*Maylandia zebra*	Cichlidae	external	developmental	Photic	RT-qPCR	laboratory
Ehlman et al. (2015)	*Poecilia reticulata*	Poecilidae	external	developmental	Photic	RT-qPCR	laboratory
Escobar-Camacho et al. (2019)	*Cichla monoculus*	Cichlidae	external	developmental	Photic	RNA-Seq	wild
Farre et al. (2023)	*Danio rerio*	Danioninae	internal	acute	T3 (386 nM)	RT-qPCR	laboratory
Novales Flamarique (2019)	*Oncorhynchus tshawytscha*	Salmonidae	external	developmental	Photic	RNA ISH	laboratory
Fogg et al. (2023)	*Acanthurus triostegus*	Acanthuridae	external	developmental	Photic	RNA-Seq	laboratory
Fogg et al. (2023)	*Acanthurus triostegus*	Acanthuridae	external	acute	Photic	RNA-Seq	laboratory
Foster et al. (2025)	*Cyprinella lutrensis*	Cyprinidae	external	developmental	Photic	RT-qPCR	wild
Frau et al. (2022)	*Solea senegalensis*	Soleidae	external	developmental	Photic	RT-qPCR	laboratory
Fuller and Claricoates (2011)	*Lucania goodei*	Fundulidae	external	acute	Photic	RT-qPCR	laboratory
Fuller et al. (2005)	*Lucania goodei*	Fundulidae	external	developmental	Photic	RT-qPCR	laboratory
Härer et al. (2017)	*Amphilophus astorquii*	Cichlidae	external	developmental	Photic	RT-qPCR	laboratory
Härer et al. (2017)	*Amphilophus citrinellus*	Cichlidae	external	developmental	Photic	RT-qPCR	laboratory
Hofmann et al. (2010)	*Labeotropheus trewavasae*	Cichlidae	external	developmental	Photic	RT-qPCR	laboratory[b]
Hofmann et al. (2010)	*Maylandia lombardoi*	Cichlidae	external	developmental	Photic	RT-qPCR	laboratory[b]
Hofmann et al. (2010)	*Maylandia mbenjii*	Cichlidae	external	developmental	Photic	RT-qPCR	laboratory[b]
Hofmann et al. (2010)	*Melanochromis auratus*	Cichlidae	external	developmental	Photic	RT-qPCR	laboratory[b]
Hofmann et al. (2010)	*Melanochromis johannii*	Cichlidae	external	developmental	Photic	RT-qPCR	laboratory[b]
Irazábal-González et al. (2024)	*Pundamilia pundamilia*	Cichlidae	external	developmental	Photic	RT-qPCR	laboratory
Iwanicki et al. (2020)	*Platichthys stellatus*	Pleuronectidae	external	acute	Photic	dPCR	laboratory

Journal of Experimental Biology • Supplementary information

Author	Species	Family	Stimulus	Exposure	Stimulus Type	Method	Site
Karagic et al. (2022)	*Amphilophus citrinellus*	Cichlidae	internal	developmental	T4 (300 µg/L)	RT-qPCR	laboratory
Kranz et al. (2018)	*Poecilia reticulata*	Poecilidae	external	developmental	Photic	dPCR	laboratory
Kranz et al. (2018)	*Poecilia reticulata*	Poecilidae	external	acute	Photic	dPCR	laboratory
Luehrmann et al. (2018)	*Ostorhinchus cyanosoma*	Apogonidae	external	developmental	Photic	RNA-Seq	laboratory
Luehrmann et al. (2018)	*Pomacentrus amboinensis*	Pomacentridae	external	developmental	Photic	RNA-Seq	laboratory
Luehrmann et al. (2018)	*Pomacentrus moluccensis*	Pomacentridae	external	developmental	Photic	RNA-Seq	laboratory
Mackin et al. (2019)	*Danio rerio*	Danioninae	internal	developmental	T3 (100 nM)	RT-qPCR	laboratory
Mackin et al. (2019)	*Danio rerio*	Danioninae	internal	acute	T3 (100 nM)	RT-qPCR	laboratory
Nandamuri et al. (2017)	*Maylandia benetos*	Cichlidae	external	acute	Photic	RT-qPCR	laboratory
Nandamuri et al. (2017)	*Maylandia mbenjii*	Cichlidae	external	acute	Photic	RT-qPCR	laboratory[b]
Sakai et al. (2016)	*Poecilia reticulata*	Poecilidae	external	developmental	Photic	RT-qPCR	laboratory
Sakai et al. (2018)	*Poecilia reticulata*	Poecilidae	external	developmental	Photic	RT-qPCR	laboratory
Schreiner et al. (2022)	*Haplochromis burtoni*	Cichlidae	external	developmental	Photic	RT-qPCR	laboratory
Schreiner et al. (2022)	*Haplochromis burtoni*	Cichlidae	internal	acute	T4 (300 µg/L)	RT-qPCR	laboratory
Schreiner et al. (2022)	*Haplochromis burtoni*	Cichlidae	internal	developmental	T4 (300 µg/L)	RT-qPCR	laboratory
Schweikert and Grace (2018)	*Megalops atlanticus*	Megalopidae	external	acute	Photic	RNA ISH	laboratory
Shao et al. (2014)	*Gasterosteus aculeatus*	Gasterosteidae	internal	acute	11KT (225 µg/L)	RT-qPCR	laboratory
Shao et al. (2014)	*Gasterosteus aculeatus*	Gasterosteidae	external	acute	Photoperiod	RT-qPCR	laboratory
Stieb et al. (2016)	*Chrysiptera rollandi*	Pomacentridae	external	developmental	Photic	RT-qPCR	wild
Stieb et al. (2016)	*Dascyllus aruanus*	Pomacentridae	external	developmental	Photic	RT-qPCR	wild
Stieb et al. (2016)	*Dascyllus reticulatus*	Pomacentridae	external	developmental	Photic	RT-qPCR	wild
Stieb et al. (2016)	*Pomacentrus amboinensis*	Pomacentridae	external	developmental	Photic	RT-qPCR	wild
Stieb et al. (2016)	*Pomacentrus coelestis*	Pomacentridae	external	developmental	Photic	RT-qPCR	wild
Stieb et al. (2016)	*Pomacentrus moluccensis*	Pomacentridae	external	developmental	Photic	RT-qPCR	wild
Stieb et al. (2016)	*Pomacentrus nagasakiensis*	Pomacentridae	external	developmental	Photic	RT-qPCR	wild
Thomson-Laing et al. (2018)	*Anguilla anguilla*	Anguillidae	internal	acute	11KT (10 µg/L)	RT-qPCR	laboratory
Torres-Dowdall et al. (2024)	*Amphilophus citrinellus*	Cichlidae	external	developmental	T4 (300 µg/L)	RNA-Seq	laboratory

Journal of Experimental Biology • Supplementary information

Author	Species	Family	Stimulus	Exposure	Stimulus Type	Method	Site
Torres-Dowdall et al. (2024)	*Amphilophus citrinellus*	Cichlidae	internal	developmental	Photic	RNA-Seq	laboratory
Valen et al. (2018)	*Gadus morhua*	Gadidae	external	developmental	Photic	RT-qPCR	laboratory
Veen et al. (2017)	*Gasterosteus aculeatus*	Gasterosteidae	external	acute	Photic	RT-qPCR	wild
Wilwert et al. (2023)	*Haplochromis sauvagei*	Cichlidae	external	developmental	Photic	RT-qPCR	laboratory
Wilwert et al. (2023)	*Pundamilia nyererei*	Cichlidae	external	developmental	Photic	RT-qPCR	laboratory
Wilwert et al. (2023)	*Pundamilia pundamilia*	Cichlidae	external	developmental	Photic	RT-qPCR	laboratory
Wright et al. (2020)	*Pundamilia nyererei*	Cichlidae	external	developmental	Photic	RT-qPCR	laboratory
Wright et al. (2020)	*Pundamilia pundamilia*	Cichlidae	external	developmental	Photic	RT-qPCR	laboratory

a. Photic is used for studies that manipulated both spectral range and light intensity simultaneously
b. Studies that compared wild and laboratory-reared individuals
Hormones used: T4 (L-Thyroxine), T3 (Triiodothyronine), 11KT (11-keto-testosterone)
Methods used: RT-qPCR (reverse transcription quantitative PCR), RNA-Seq (RNA sequencing), RNA ISH (RNA *in situ* hybridization), dPCR (digital PCR)

Journal of Experimental Biology • Supplementary information

Table S2. Summary statistics of mixed-effect meta-analytical models.

	beta	± 95% CI	Q_E	df	*P*-value	I^2	I^2 study	I^2 experiment / study	I^2 species	I^2 phylogeny
Overall	0.949	0.196	1570.6	569	<.0001	57.71	48.62	3.61	5.47	0
Q_M	-	-	134.8	4	<.0001	-	-	-	-	-
sws1	1.285	0.344	383.168	87	< .0001	70.75	27.86	9.07	33.82	0
sws2	0.91	0.442	461.789	149	< .0001	74.66	53.05	11.2	0	10.4
rh2	0.861	0.157	452.369	191	< .0001	42.01	0	25.02	16.99	0
lws	0.972	0.259	305.96	127	< .0001	68.91	57.75	4.65	6.52	0
rh1	0.877	0.898	33.84	14	0.002	63.82	-	-	-	-
Internal	1.694	0.504	228.70	83	< .0001	24.96	1.65	11.7	0	11.61
sws1	1.376	0.627	35.62	13	< .0001	24.41	0	0	24.41	0
sws2	1.957	1.104	81.03	22	< .0001	60.06	0	50.03	0	10.02
rh2	1.345	0.335	59.29	24	< .0001	13.39	0	0	0	3.39
lws	2.436	0.913	39.30	19	0.004	56.94	0	56.94	0	0
rh1	2.273	1.75	2.68	0.101	> .1	-	-	-	-	-
External	0.839	0.188	1351.799	488	< .0001	53.79	44.79	1.2	7.79	0
sws1	1.298	0.365	344.518	73	< .0001	74.14	25.1	16.82	32.22	0
sws2	0.806	0.456	334.098	126	< .0001	73.07	56.06	0.31	0	16.71
rh2	0.797	0.162	377.425	166	< .0001	41.83	5.84	26.52	9.47	0
lws	0.827	0.22	232.521	107	< .0001	58.25	40.83	5.94	11.47	0
rh1	0.44	0.263	21.667	12	> .1	0	0	0	0	0
Developmental	0.909	0.184	1303.408	425	< .0001	47.33	3.72	0	43.61	0
sws1	1.456	0.517	305.113	64	< .0001	76.51	0.03	4.6	65.6	6.29
sws2	0.924	0.602	389.638	109	< .0001	76.92	53.65	1.65	0	21.61
rh2	0.831	0.183	354.44	145	< .0001	43.29	0	21.82	21.47	0
lws	0.839	0.208	174.87	93	< .0001	44.76	0	5.74	39.02	0
rh1	0.432	0.273	20.683	10	> .1	-	-	-	-	-
Acute	1.233	0.395	365.98	146	< .0001	73.2	72.48	0	0.72	0
sws1	1.058	0.462	53.20	22	< .0001	65.7	65.7	0	0	0
sws2	1.037	0.544	72.13	39	0.001	75.08	75.08	0	0	0
rh2	0.961	0.31	97.60	45	< .0001	48.56	25.71	22.85	0	0
lws	1.711	0.806	130.13	33	< .0001	92.38	89.67	0	2.72	0
rh1	1.255	1.334	8.20	3	0.042	59.01	-	-	-	-

Table S3. Sample sizes for each opsin across experimental designs

		sws1	*sws2*	*rh2*	*lws*	*rh1*
**Internal**						
	Developmental	11	18	14	15	0
	Acute	3	5	11	5	2
**External**						
	Developmental	54	92	132	79	11
	Acute	20	35	35	29	2

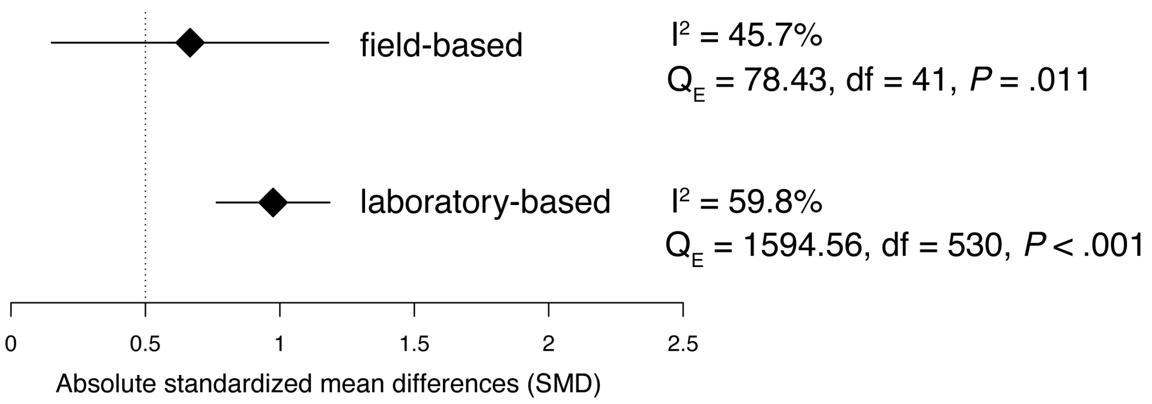

Fig. S1. Mean estimate and 95% confidence intervals of absolute standardized mean differences between laboratory- and field-based studies.

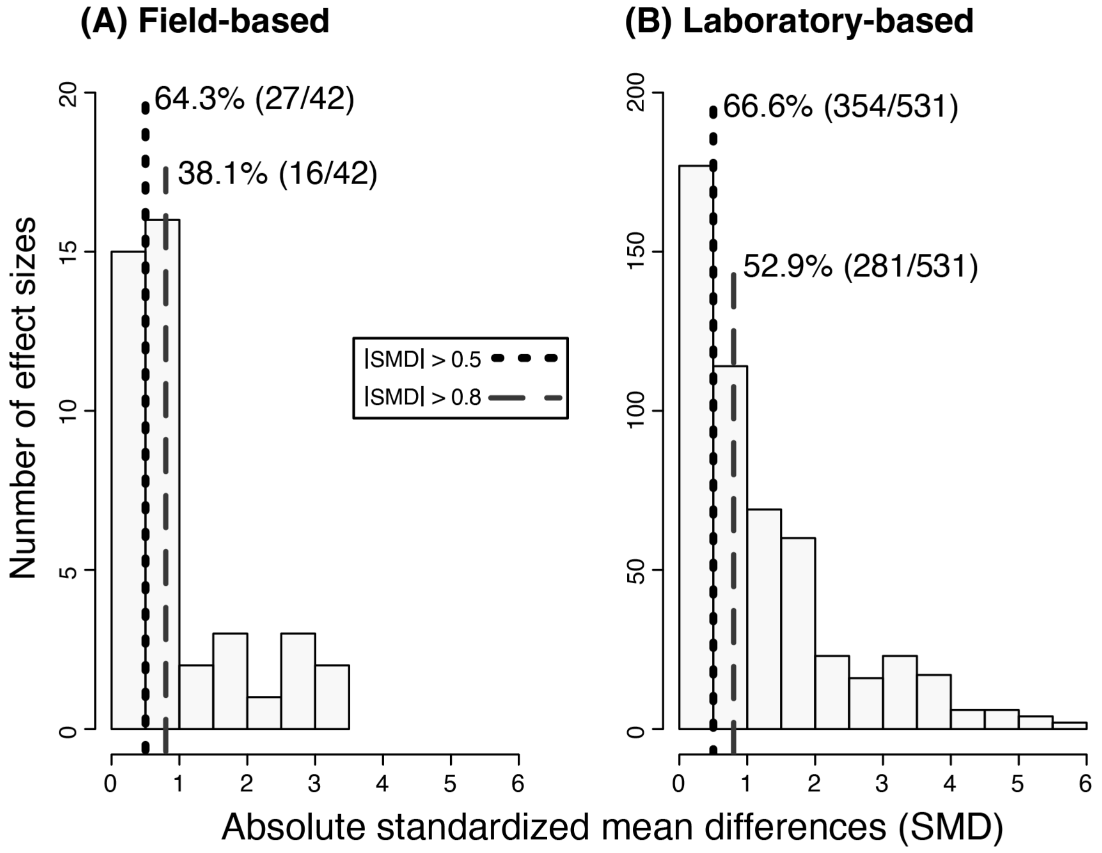

Fig. S2. Histogram of absolute standardized mean differences (SMD) of opsin gene expression plasticity in teleost fish between laboratory- and field-based studies. Dashed line shows the thresholds above which effect sizes are considered moderate (SMD > 0.5) or strong (SMD > 0.8). Percentages show the proportion of effect sizes above each threshold.

Journal of Experimental Biology • Supplementary information

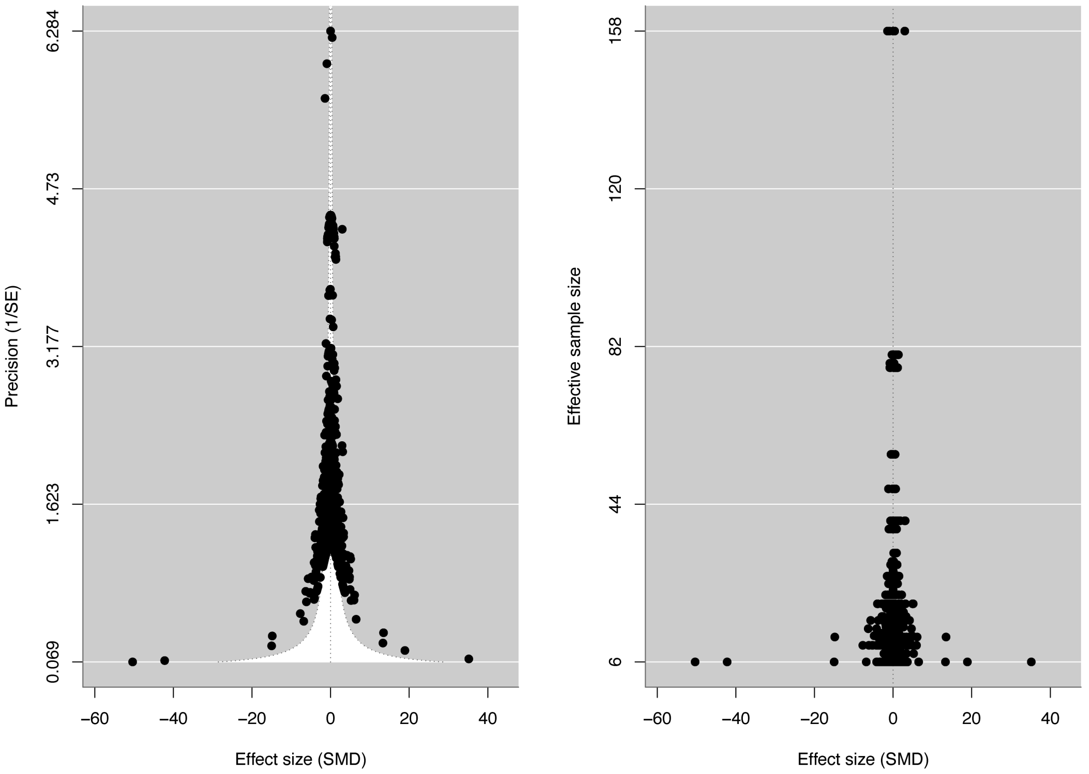

Fig. S3. Funnel plots displaying the raw standardized mean differences (SMD) relative to the precision, 1/SE (left) and the effective sample size (right). No clear asymmetric distribution is observed among effect sizes suggesting little evidence for publication bias. However, funnel plots might not be adequate for ecological and evolutionary meta-analyses given their high degree of heterogeneity (Nakagawa et al., 2022). For a formal test of publication bias see Fig. S4.

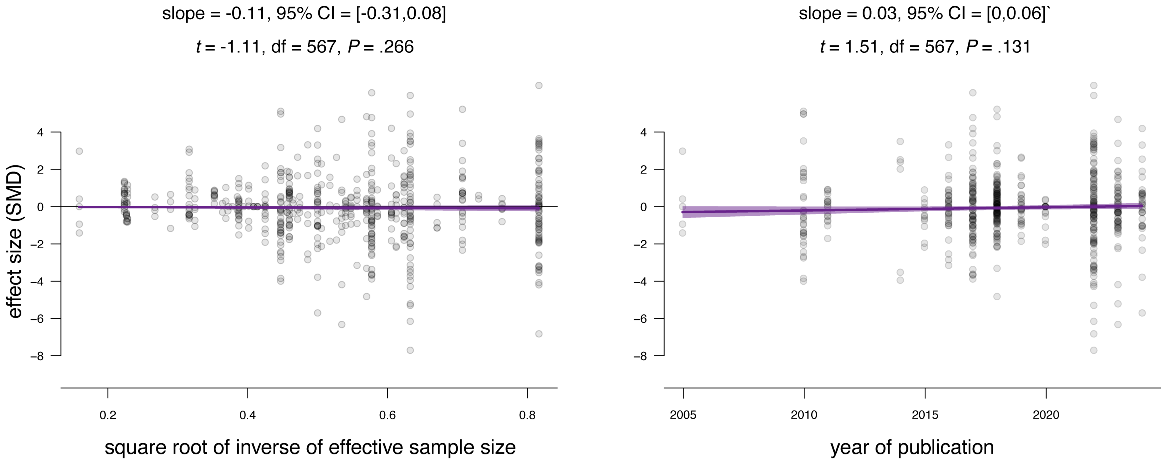

Fig. S4. Multi-moderator meta-regression suggest no significant publication bias due to small-study effects (left) or decline effect over time (right). Solid line represents the model estimate and the shading shows its 95% confidence intervals. Modelling approach as recommended by Nakagawa et al. (2022). The lack of a clear trend between moderators SE and year of publication with effect sizes suggests that such bias in the literature do not explain the differences in opsin gene expression plasticity reported in this meta-analysis.

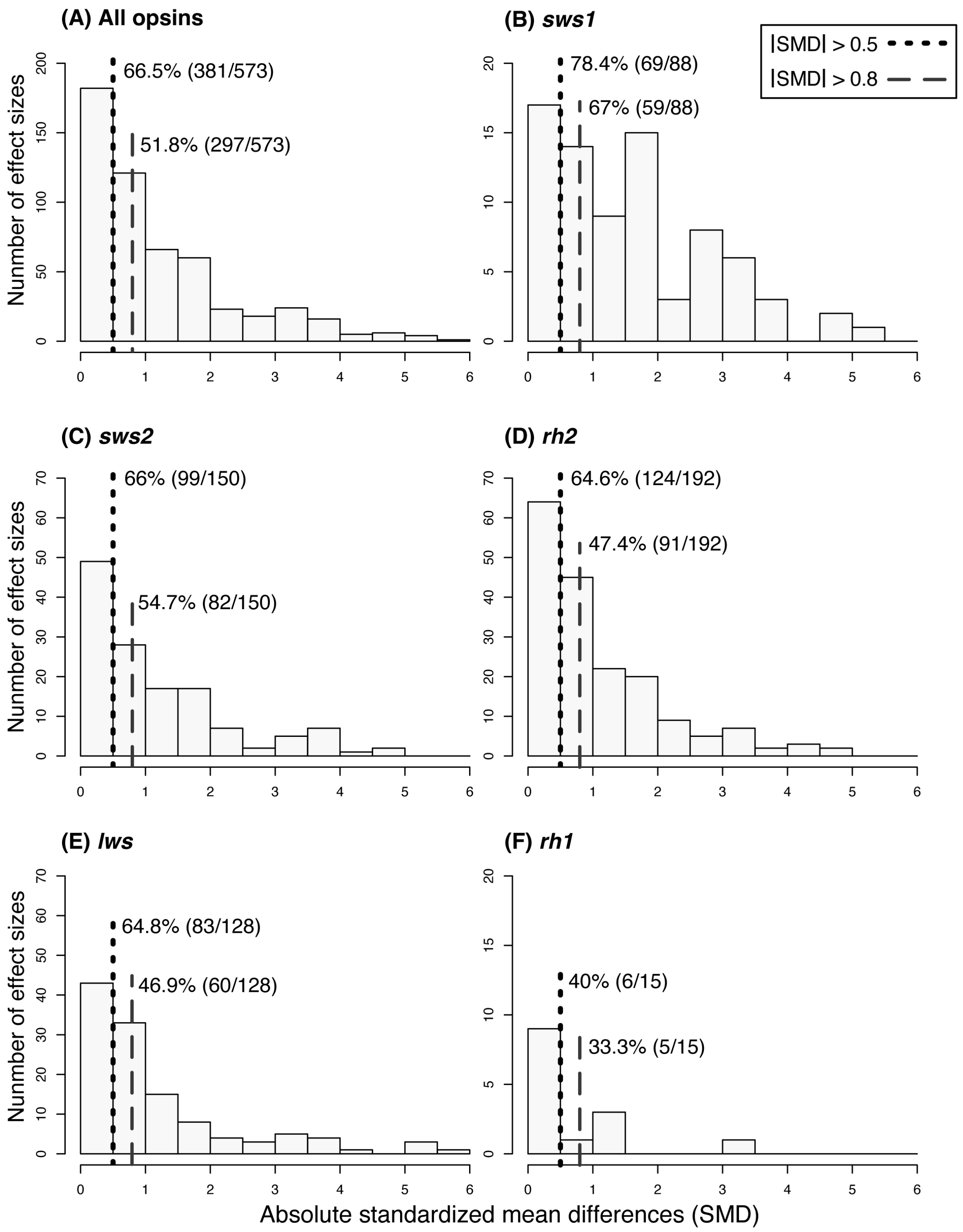

Fig. S5. Histogram of absolute standardized mean differences (SMD) of opsin gene expression plasticity among teleost fish. Dashed line shoe the thresholds above which effect sizes are considered moderate (SMD > 0.5) or strong (SMD > 0.8). Percentages show the proportion of effect sizes above each threshold.

Journal of Experimental Biology • Supplementary information

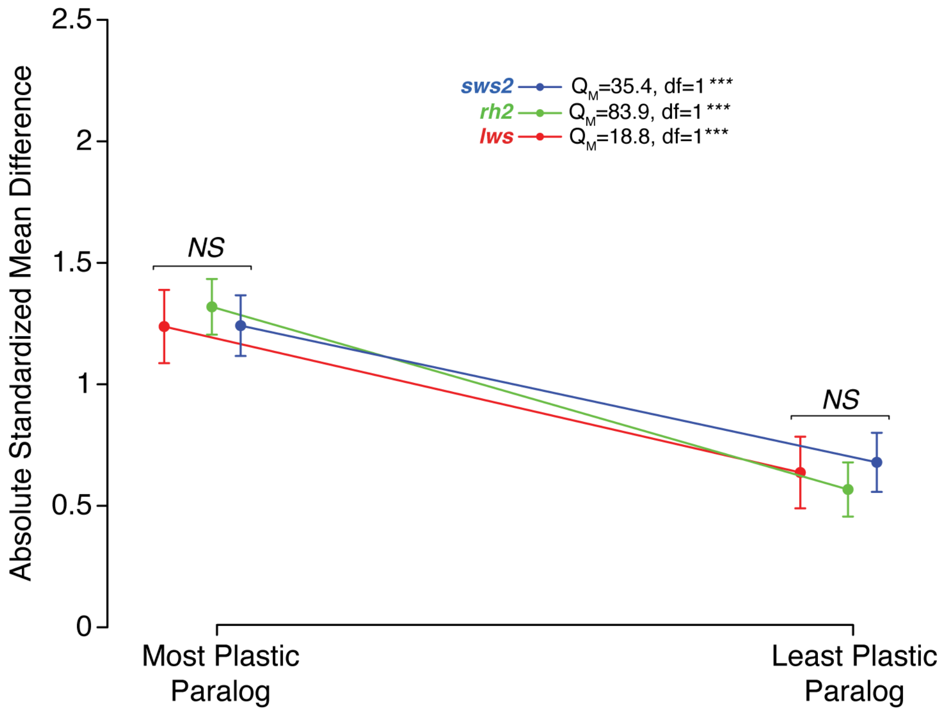

Fig. S6. Gene expression plasticity of the most and least plastic paralog within each opsin gene class. Opsin gene class *sws1* and *rh1* did not contain paralogs within our dataset. Dot and error bars represent mean and 95% confidence intervals. *P*-values estimated using Wald-type chi-square test and adjusted for multiple comparisons using false-discovery rate. *** = $P < .001$, *NS* = $P > .05$

Journal of Experimental Biology • Supplementary information

Opsin gene expression plasticity

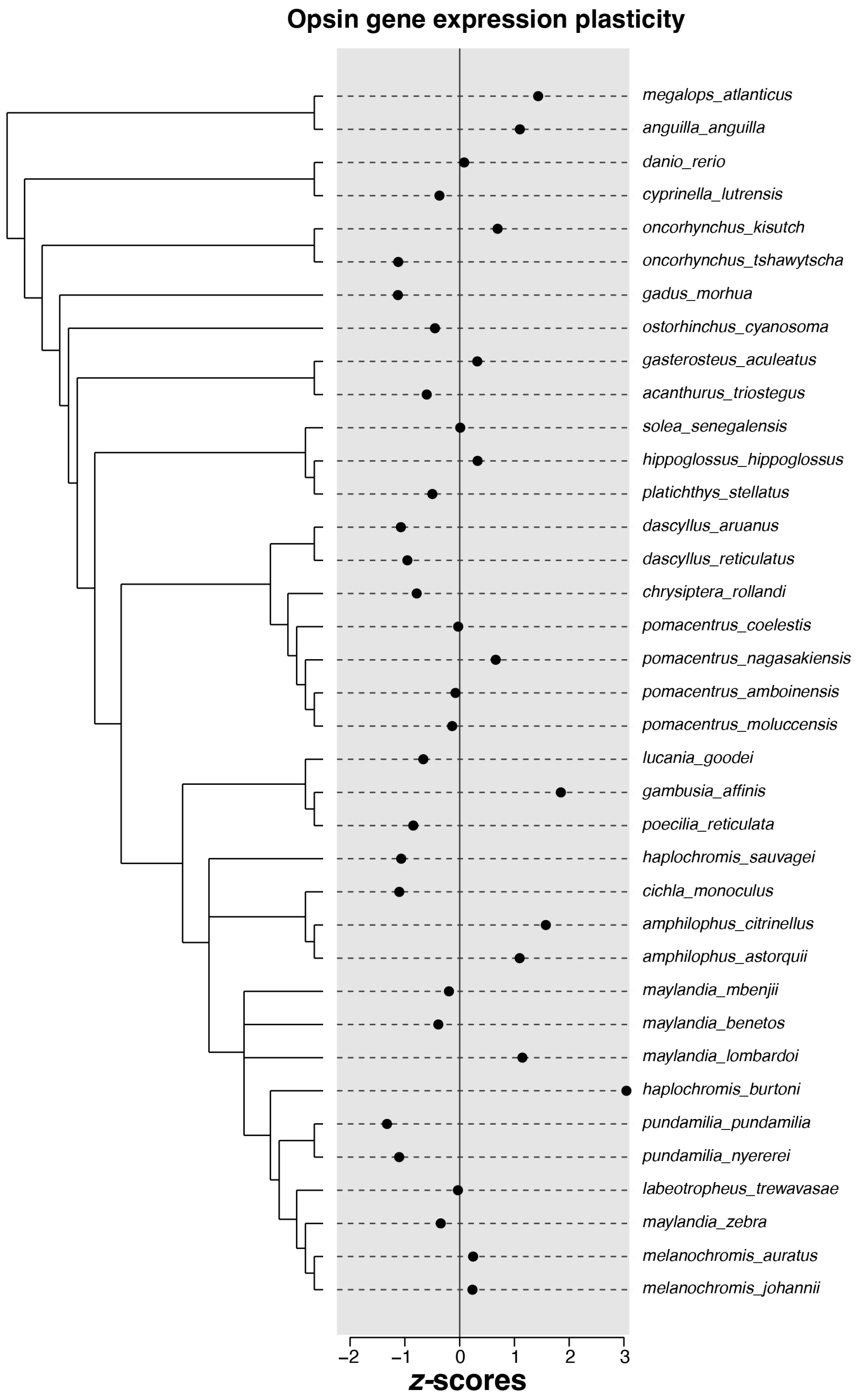

Fig. S7. Phylogenetic tree used to estimate the phylogenetic signal of opsin gene expression plasticity (z-transformed). Dots represent the raw mean opsin gene plasticity for each species reported in the literature. Phylogeny based on Open Tree of Life (Hinchliff et al., 2015).

Journal of Experimental Biology • Supplementary information

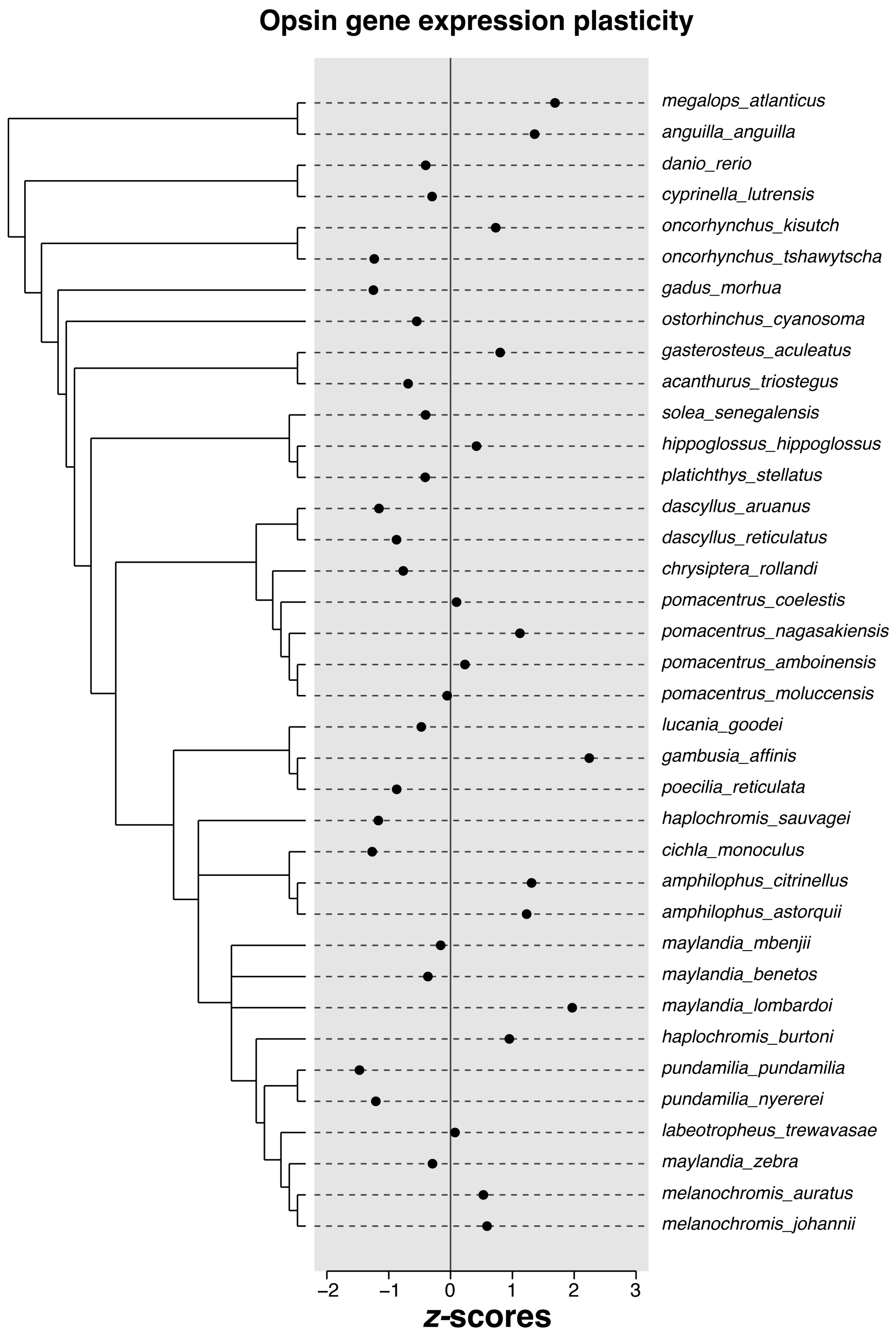

Fig. S8. Phylogenetic tree showing the phylogenetic signal of opsin gene expression plasticity (z-transformed). Dots represent the mean estimates of opsin gene expression plasticity based on meta-analysis model accounting for heterogeneity and phylogenetic relationship (Table S2). Phylogeny based on Open Tree of Life (Hinchliff et al., 2015).

Journal of Experimental Biology • Supplementary information

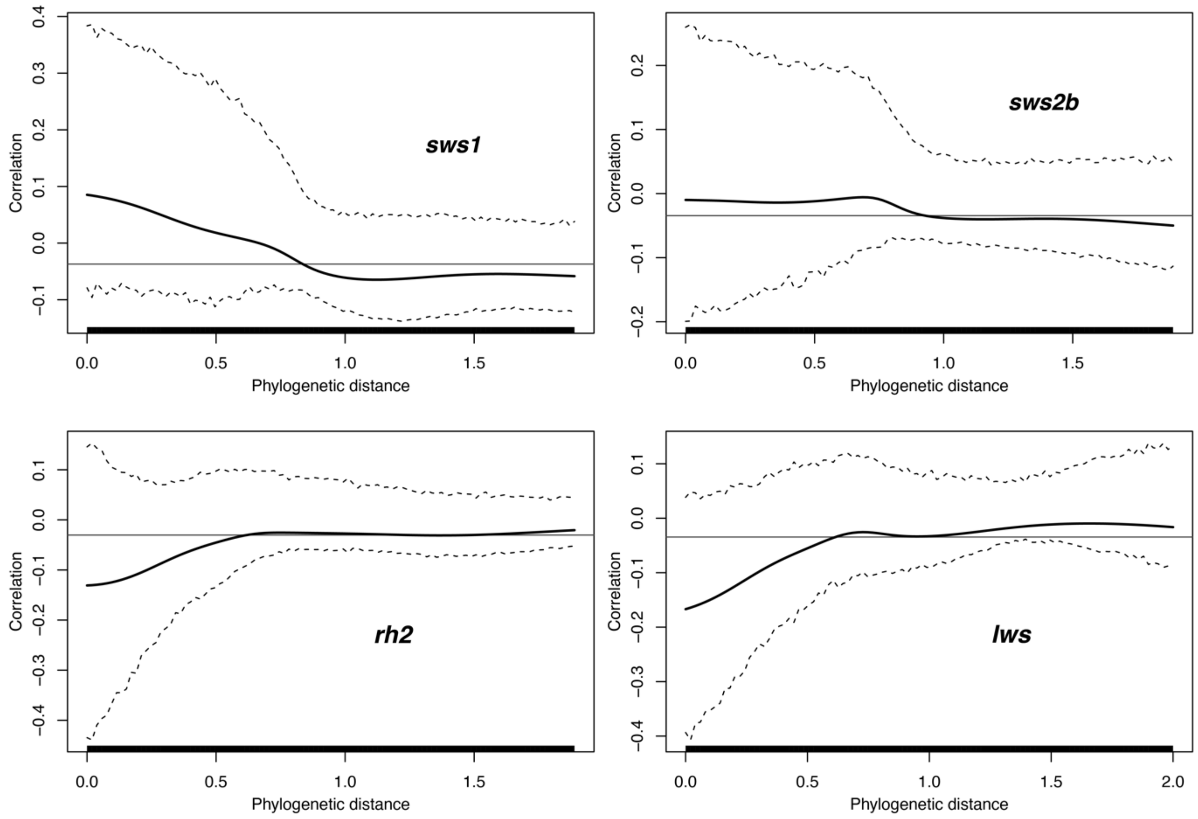

Fig. S9. Phylogenetic signal of opsin gene expression plasticity for *sws1, sws2, rh2* and *lws* visual cone opsins. Due to a lack of sampling across the phylogeny, *rh1* could not be estimated. In all cases, overall Pagel's λ was smaller than 0.05 suggesting phylogeny-independent evolution of the trait. Phylocorrelograms were generated using the *phylosignal* package in R (Keck et al., 2016).

Journal of Experimental Biology • Supplementary information

References

Bakker, T. C. M., Hiermes, M., Müller, B., Martin, S., Rennison, D. J. and Rick, I. P. (2024). Adaptive variation in opsin expression of sticklebacks from different photic habitats. *Hydrobiologia*.

Bolstad, K. and Novales Flamarique, I. (2023). Two mechanisms of retinal photoreceptor plasticity underlie rapid adaptation to novel light environments. *Journal of Comparative Neurology* **531**, 1080-1094.

Chang, C.-H. (2023). Rapid adjustment of cone opsin expression profiles may help Western mosquitofish (*Gambusia affinis*) maintain foraging efficiency in distinct light environments. *Hydrobiologia* **850**, 1059-1071.

Chang, C.-H. and Yan, H. Y. (2019). Plasticity of opsin gene expression in the adult red shiner (*Cyprinella lutrensis*) in response to turbid habitats. *PloS One* **14**, e0215376.

Dalton, B. E., Lu, J., Leips, J., Cronin, T. W. and Carleton, K. L. (2015). Variable light environments induce plastic spectral tuning by regional opsin coexpression in the African cichlid fish, *Metriaclima zebra*. *Molecular Ecology* **24**, 4193-4204.

Ehlman, S. M., Sandkam, B. A., Breden, F. and Sih, A. (2015). Developmental plasticity in vision and behavior may help guppies overcome increased turbidity. *Journal of Comparative Physiology A* **201**, 1125-1135.

Escobar-Camacho, D., Pierotti, M. E. R., Ferenc, V., Sharpe, D. M. T., Ramos, E., Martins, C. and Carleton, K. L. (2019). Variable vision in variable environments: the visual system of an invasive cichlid (*Cichla monoculus*, Agassiz, 1831) in Lake Gatun, Panama. *Journal of Experimental Biology* **222**, jeb188300.

Farre, A. A., Thomas, P., Huang, J., Poulsen, R. A., Owusu Poku, E. and Stenkamp, D. L. (2023). Plasticity of cone photoreceptors in adult zebrafish revealed by thyroid hormone exposure. *Scientific Reports* **13**, 15697.

Fogg, L. G., Cortesi, F., Gache, C., Lecchini, D., Marshall, N. J. and de Busserolles, F. (2023). Developing and adult reef fish show rapid light-induced plasticity in their visual system. *Molecular Ecology* **32**, 167-181.

Foster, T. N., Williamson, A. G., Foster, B. R. and Toomey, M. B. (2025). Light environment and seasonal variation in the visual system of the red shiner (*Cyprinella lutrensis*). *Journal of Experimental Biology* **228**.

Frau, S., Paullada-Salmerón, J. A., Paradiso, I., Cowan, M. E., Martín-Robles, Á. J. and Muñoz-Cueto, J. A. (2022). From Embryo to Adult Life: Differential Expression of Visual Opsins in the Flatfish *Solea senegalensis* Under Different Light Spectra and Photoperiods. *Frontiers in Marine Science* **9**.

Fuller, R. C., Carleton, K. L., Fadool, J. M., Spady, T. C. and Travis, J. (2005). Genetic and environmental variation in the visual properties of bluefin killifish, *Lucania goodei*. *Journal of Evolutionary Biology* **18**, 516-523.

Fuller, R. C. and Claricoates, K. M. (2011). Rapid light-induced shifts in opsin expression: finding new opsins, discerning mechanisms of change, and implications for visual sensitivity. *Molecular Ecology* **20**, 3321-3335.

Gan, K. J. and Novales Flamarique, I. (2010). Thyroid hormone accelerates opsin expression during early photoreceptor differentiation and induces opsin switching in differentiated TRα-expressing cones of the salmonid retina. *Developmental Dynamics* **239**, 2700-2713.

Härer, A., Torres-Dowdall, J. and Meyer, A. (2017). Rapid adaptation to a novel light environment: The importance of ontogeny and phenotypic plasticity in shaping the visual system of Nicaraguan Midas cichlid fish (*Amphilophus citrinellus* spp.). *Molecular Ecology* **26**, 5582-5593.

Journal of Experimental Biology • Supplementary information

Hinchliff, C. E., Smith, S. A., Allman, J. F., Burleigh, J. G., Chaudhary, R., Coghill, L. M., Crandall, K. A., Deng, J., Drew, B. T., Gazis, R. et al. (2015). Synthesis of phylogeny and taxonomy into a comprehensive tree of life. *Proceedings of the National Academy of Sciences* **112**, 12764-12769.

Hofmann, C. M., O'Quin, K. E., Smith, A. R. and Carleton, K. L. (2010). Plasticity of opsin gene expression in cichlids from Lake Malawi. *Molecular Ecology* **19**, 2064-2074.

Irazábal-González, L., Wright, D. S. and Maan, M. E. (2024). Developmental and environmental plasticity in opsin gene expression in Lake Victoria cichlid fish. *Evolution & Development* **26**, e12465.

Iwanicki, T., Haman, C., Liu, A. and Taylor, J. S. (2020). Light induced changes in starry flounder (*Platichthys stellatus*) opsin expression and its influence on vision estimated from a camouflage-based behavioural assay. *bioRxiv*, 2020.07.30.228627.

Karagic, N., Härer, A., Meyer, A. and Torres-Dowdall, J. (2022). Thyroid hormone tinkering elicits integrated phenotypic changes potentially explaining rapid adaptation of color vision in cichlid fish. *Evolution* **76**, 837-845.

Keck, F., Rimet, F., Bouchez, A. and Franc, A. (2016). phylosignal: an R package to measure, test, and explore the phylogenetic signal. *Ecology and Evolution* **6**, 2774-2780.

Kranz, A. M., Forgan, L. G., Cole, G. L. and Endler, J. A. (2018). Light environment change induces differential expression of guppy opsins in a multi-generational evolution experiment. *Evolution* **72**, 1656-1676.

Luehrmann, M., Stieb, S. M., Carleton, K. L., Pietzker, A., Cheney, K. L. and Marshall, N. J. (2018). Short-term colour vision plasticity on the reef: changes in opsin expression under varying light conditions differ between ecologically distinct fish species. *Journal of Experimental Biology* **221**.

Mackin, R. D., Frey, R. A., Gutierrez, C., Farre, A. A., Kawamura, S., Mitchell, D. M. and Stenkamp, D. L. (2019). Endocrine regulation of multichromatic color vision. *Proceedings of the National Academy of Sciences* **116**, 16882-16891.

Nakagawa, S., Lagisz, M., Jennions, M. D., Koricheva, J., Noble, D. W. A., Parker, T. H., Sánchez-Tójar, A., Yang, Y. and O'Dea, R. E. (2022). Methods for testing publication bias in ecological and evolutionary meta-analyses. *Methods in Ecology and Evolution* **13**, 4-21.

Nandamuri, S. P., Yourick, M. R. and Carleton, K. L. (2017). Adult plasticity in African cichlids: Rapid changes in opsin expression in response to environmental light differences. *Molecular Ecology* **26**, 6036-6052.

Novales Flamarique, I. (2019). Light exposure during embryonic and yolk-sac alevin development of Chinook salmon *Oncorhynchus tshawytscha* does not alter the spectral phenotype of photoreceptors. *Journal of Fish Biology* **95**, 214-221.

Sakai, Y., Kawamura, S. and Kawata, M. (2018). Genetic and plastic variation in opsin gene expression, light sensitivity, and female response to visual signals in the guppy. *Proceedings of the National Academy of Sciences* **115**, 12247-12252.

Sakai, Y., Ohtsuki, H., Kasagi, S., Kawamura, S. and Kawata, M. (2016). Effects of light environment during growth on the expression of cone opsin genes and behavioral spectral sensitivities in guppies (*Poecilia reticulata*). *BMC Evolutionary Biology* **16**, 106.

Schreiner, M., Yourick, M., Juntti, S. and Carleton, K. (2022). Environmental plasticity in opsin expression due to light and thyroid hormone in adult and developing *Astatotilapia burtoni*. *Hydrobiologia* **850**.

Schweikert, L. E. and Grace, M. S. (2018). Altered environmental light drives retinal change in the Atlantic Tarpon (*Megalops atlanticus*) over timescales relevant to marine environmental disturbance. *BMC Ecology* **18**, 1.

Shao, Y. T., Wang, F.-Y., Fu, W.-C., Yan, H. Y., Anraku, K., Chen, I. S. and Borg, B. (2014). Androgens Increase lws Opsin Expression and Red Sensitivity in Male Three-Spined Sticklebacks. *PloS One* **9**, e100330.

Stieb, S. M., Carleton, K. L., Cortesi, F., Marshall, N. J. and Salzburger, W. (2016). Depth-dependent plasticity in opsin gene expression varies between damselfish (Pomacentridae) species. *Molecular Ecology* **25**, 3645-3661.

Thomson-Laing, G., Jasoni, C. L. and Lokman, P. M. (2018). The effects of migratory stage and 11-ketotestosterone on the expression of rod opsin genes in the shortfinned eel (*Anguilla australis*). *General and Comparative Endocrinology* **257**, 211-219.

Torres-Dowdall, J., Karagic, N., Prabhukumar, F. and Meyer, A. (2024). Differential Regulation of Opsin Gene Expression in Response to Internal and External Stimuli. *Genome Biology and Evolution* **16**.

Valen, R., Karlsen, R. and Helvik, J. V. (2018). Environmental, population and life-stage plasticity in the visual system of Atlantic cod. *Journal of Experimental Biology* **221**.

Veen, T., Brock, C., Rennison, D. and Bolnick, D. (2017). Plasticity contributes to a fine-scale depth gradient in sticklebacks' visual system. *Molecular Ecology* **26**, 4339-4350.

Wilwert, E., Etienne, R. S., Van De Zande, L. and Maan, M. E. (2023). Visual system plasticity is differently mediated by cone opsin expression and chromophore composition in closely related cichlid species. *Hydrobiologia* **850**, 2299-2314.

Wright, D. S., van Eijk, R., Schuart, L., Seehausen, O., Groothuis, T. G. G. and Maan, M. E. (2020). Testing sensory drive speciation in cichlid fish: Linking light conditions to opsin expression, opsin genotype and female mate preference. *Journal of Evolutionary Biology* **33**, 422-434.
